# Intense Exercise and Aerobic Conditioning Associated with Chromium or L-Carnitine Supplementation Modified the Fecal Microbiota of Fillies

**DOI:** 10.1371/journal.pone.0167108

**Published:** 2016-12-09

**Authors:** Maria Luiza Mendes de Almeida, Walter Heinz Feringer, Júlia Ribeiro Garcia Carvalho, Isadora Mestriner Rodrigues, Lilian Rezende Jordão, Mayara Gonçalves Fonseca, Adalgiza Souza Carneiro de Rezende, Antonio de Queiroz Neto, J. Scott Weese, Márcio Carvalho da Costa, Eliana Gertrudes de Macedo Lemos, Guilherme de Camargo Ferraz

**Affiliations:** 1 Department of Technology, Faculdades de Ciências Agrárias e Veterinárias, UNESP Univ Estadual Paulista, Laboratório de Bioquímica de Microrganismos e Plantas, Jaboticabal, São Paulo, Brazil; 2 Department of Animal Morphology and Physiology, Faculdades de Ciências Agrárias e Veterinárias, UNESP Univ Estadual Paulista, Laboratório de Farmacologia e Fisiologia do Exercício Equino (LAFEQ), Jaboticabal, São Paulo, Brazil; 3 Department of Animal Sciences, Escola de Veterinária, Universidade Federal de Minas Gerais, Belo Horizonte, Minas Gerais, Brazil; 4 Department of Pathobiology, Ontario Veterinary College, University of Guelph, Ontario, Canada; Max Rubner-Institut, GERMANY

## Abstract

Recent studies performed in humans and rats have reported that exercise can alter the intestinal microbiota. Athletic horses perform intense exercise regularly, but studies characterizing horse microbiome during aerobic conditioning programs are still limited. Evidence has indicated that this microbial community is involved in the metabolic homeostasis of the host. Research on ergogenic substances using new sequencing technologies have been limited to the intestinal microbiota and there is a considerable demand for scientific studies that verify the effectiveness of these supplements in horses. L-carnitine and chromium are potentially ergogenic substances for athletic humans and horses since they are possibly able to modify the metabolism of carbohydrates and lipids. This study aimed to assess the impact of acute exercise and aerobic conditioning, associated either with L-carnitine or chromium supplementation, on the intestinal microbiota of fillies. Twelve “Mangalarga Marchador” fillies in the incipient fitness stage were distributed into four groups: control (no exercise), exercise, L-carnitine (10g/day) and chelated chromium (10mg/day). In order to investigate the impact of acute exercise or aerobic conditioning on fecal microbiota all fillies undergoing the conditioning program were analyzed as a separate treatment. The fillies underwent two incremental exercise tests before and after training on a treadmill for 42 days at 70–80% of the lactate threshold intensity. Fecal samples were obtained before and 48 h after acute exercise (incremental exercise test). Bacterial populations were characterized by sequencing the V4 region of the 16S rRNA gene using the MiSeq Illumina platform, and 5,224,389 sequences were obtained from 48 samples. The results showed that, overall, the two most abundant phyla were Firmicutes (50.22%) followed by Verrucomicrobia (15.13%). The taxa with the highest relative abundances were unclassified Clostridiales (17.06%) and "5 genus *incertae sedis*" from the phylum Verrucomicrobia (12.98%). There was a decrease in the phylum Chlamydiae and in the genus *Mycobacterium* after the second incremental exercise test. Intense exercise changed the community’s structure and aerobic conditioning was associated with changes in the composition and structure of the intestinal bacterial population of fillies. The intra-group comparison showed that chromium or L-carnitine induced moderate changes in the fecal microbiota of fillies, but the microbiota did not differ from the control group, which was exercised with no supplementation. Fecal pH correlated positively with Simpson’s index, while plasma pH correlated negatively. Our results show that exercise and aerobic conditioning can change in the microbiota and provide a basis for further studies enrolling a larger number of horses at different fitness levels to better understand the effects of exercise and training on the intestinal microbiota of horses.

## Introduction

The intestinal microbiota is a complex polymicrobial environment involving close interactions between the host and microorganisms, particularly bacteria. In horses, the intestinal microbiota can be modified by various factors, such as gastrointestinal diseases [1], changes in diet [2], the use of antibiotics [3,4] and general anesthesia [5]. A recent study found that changes in the intestinal microbiota might predict the development of postpartum gastrointestinal disease [6], indicating that alterations in the microbiota resulting from various physiological events might alter the risk of subsequent disease. Recent studies using genetic sequencing techniques reported that exercise modified the structure of the intestinal microbiota in mice [7,8,9,10]. However, there is little information in the literature regarding athlete horses, a term that describes horses undergoing a practice of intense exercise and conditioning programs, and possible changes in their intestinal microbiota.

Many horses routinely perform intense exercise (exercise-induced metabolic acidosis by increasing muscle production of hydrogen ions). If one considers exercise a stimulus that alters homeostasis, a single effort session can promote reversible changes in several physiological variables, and these alterations can be confirmed by quantifying clinical laboratory variables. Depending on the effort as well as the duration and intensity of the exercise session, these changes can be observed for physiological variables in both humans and horses [11,12]. For example, the systemic, reversible, increased glycolytic rate and hydrolysis of ATP during the contraction of skeletal muscle fibers with the induction of metabolic acidosis, associated with increased plasma lactate production, is the classical response to the intense incremental exercise test [12]. In addition, monitoring the activity of serum enzymes such as creatine kinase (CK) and aspartate aminotransferase (AST) after and during acute effort in conditioning programs may reveal changes in the permeability of the sarcolemma. These enzymes are considered biomarkers of the functional state of the muscle fiber [13].

A recent study examining the impact of exercise associated with varied dietary intake identified increased intestinal microbiota diversity and a positive correlation with CK activity in high-performance rugby athletes [14,15]. However, corresponding data for horses are lacking despite the commonality of intense exercise and gastrointestinal diseases in both species.

Many oral supplements are marketed for human and equine athletes as a means to improve athletic performance, often with little evidence or a clear mechanism. Chromium (Chr) and L-carnitine (LC) are potential ergogenic substances for athletes that can increase the energy available for exercise and delay the onset of fatigue [16,17]. Chronic oral supplementation with Chr or LC during an aerobic conditioning program produced moderate changes in the energy metabolism biomarkers of fillies [18]. Whether supplements such as these also alter the microbiota or exert exercise-associated microbiota changes is unclear.

Thus, the objectives of this study were to investigate the impact of intense exercise (incremental exercise testing) and an aerobic conditioning program on the intestinal microbiota in fillies and to assess the effects of supplementation with Chr or LC on exercise- and aerobic conditioning-associated microbiota alterations.

## Material and Methods

The study followed the Ethical Principles in Animal Experimentation adopted by the Brazilian College of Animal Experimentation and approved by the Ethics Committee on Animal Use (CEUA–FCAV/Univ Estadual Paulista) under protocol 016842/13.

### Animals and adaptation period

Twelve Mangalarga Marchador fillies (MM), 2.5 to 3 years old, with an average body weight of 330 ± 30 kg and an incipient stage of fitness underwent an adjustment period that lasted 34 days in the Laboratory of Pharmacology and Equine Exercise Physiology (Univ Estadual Paulista/FCAV/DMFA/LAFEQ). During this period, the fillies were acclimatized to the research facility and accustomed to the management, diet, training facilities, and equipment: a treadmill (Galloper treadmill, Sahinco LTDA, Palmital, Brazil) and an automatic walker (Galloper treadmill, Sahinco LTDA, Palmital, Brazil). In the first five days, the animals were dewormed (Equimax—Virbac Saúde Animal, Jurubatuba, São Paulo, Brazil) and underwent routine management procedures such as hoof trimming and dental assessment. No animals received antibiotics or anti-inflammatory drugs, and gastrointestinal disease had not been reported in the previous 6-month period. All fillies were housed in paddocks with pastures.

### Diet

Diet consisted of a concentrate at 2.5% of body weight daily, based on dry matter [19] and roughage (approximately 4 kg of Tifton 85 hay and *Panicum maximum* cv. Massai pasture) (Table 1). The concentrate consisted of 68% corn, 12% soybean meal, 15% wheat bran, 3% calcitic lime, 1% dicalcium phosphate and 1% mineral salt (Coequi Plus^®^), as well as 40 mL of soybean oil per day. In addition, mineral salt (Coequi Plus–Tortuga, São Paulo, Brazil) and water were provided freely.

**Table 1:** Percentage of crude protein (CP), neutral (NDF) and acid (ADF) detergent fiber, mineral matter (MM), calcium (Ca), phosphorus (P), digestible energy (DE), total digestible nutrients (TDN) based on dry matter (DM) and in Mcal/kg of feed supplied during the trial.

Feed	DM (%)	CP (%)	NDF (%)	ADF (%)	MM (%)	Ca (%)	P (%)	DE Mcal/Kg	TDN (%)
**Pasture *Panicum maximum* cv. Massai**	31.4	11.8	80.7	37.5	7.45	0.42	0.37	-	59.5
**Hay Tifton 85 (*Cynodon* spp.)**	94.9	9.4	80.7	39.9	10.02	0.48	0.27	2.50	56.0
**Concentrate**	90.2	14.2	13.9	5.4	8.00	1.32	0.55	3.15	-
Mineral salt*	100.0	-	-	-	100.00	17.5	6.0	-	-

*Composition per kg of mineral salt: Calcium 175 g; Phosphorus 60 g; Magnesium 13.6 g; Sulfur 12 g; Sodium 120 g; Zinc 2.200 mg; Copper 1.200 mg; Manganese 970 mg; Iron 2.000 mg; Cobalt 21 mg; Iodine 125 mg; Selenium 10 mg; Fluor (max.) 600 mg.

Three fillies were supplemented with 10 g per day of L-carnitine powder (SweetMix^®^, Sorocaba, São Paulo, Brazil), and three animals were supplemented with 10 mg per day of chelated chromium (Tortuga, São Paulo, Brazil), both of which were offered with the concentrate. Body weight was determined using a digital scale before the first incremental exercise testing (IET) and on days 14, 32 and immediately before the second IET.

### Incremental exercise testing

The incremental exercise testing consisted of a 10-minute warm-up (5 min at 1.7 m s^-1^ and 5 min at 3.5 m s^-1^ without inclination) [18]. After the warm-up, the treadmill was raised to a 3% slope at a speed of 4 m s^-1^, and the progressive speed phase began, during which speed was increased by 1 m s^-1^ every three minutes. Exercise continued until the animals reached fatigue, which was identified by the fillies’ inability to follow the belt speed even under voice command (incentive) to continue (fatigue). The fatigue was defined as the moment in which a horse could no longer keep up with the treadmill despite humane encouragement. The cooldown lasted 10 minutes (5 min at 3.5 m s^-1^ and 5 min at 1.7 m s^-1^). Blood samples were collected during the last 30 seconds of each stage through a catheter (BD^™^ Insyte 14 GA, Chácara Santo Antonio, Brazil) and wand system inserted into the external jugular vein. Plasma lactate concentration vs. velocity stage data were used to determine the lactate threshold (LT).

### Conditioning protocol

**Fig 1:**
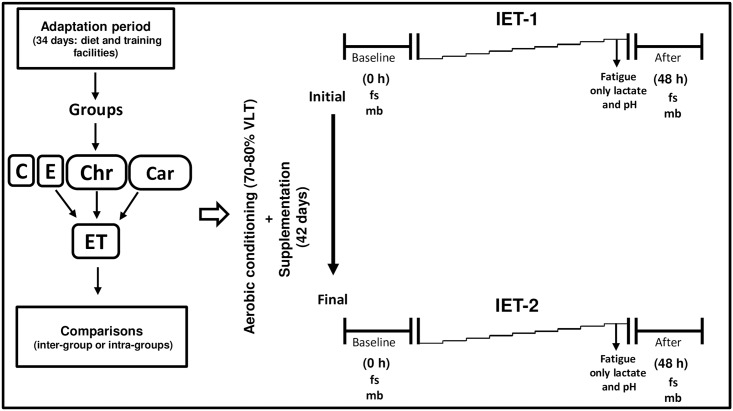
Workflow of the experimental protocol. C (control group, did not exercise); E = only conditioning; Car = conditioning plus L-carnitine supplementation; Chr = conditioning plus chromium supplementation; ET (exercise total: all fillies undergoing the conditioning were analyzed as a separate treatment). IET, incremental exercise test (IET-1 and IET-2, before and after aerobic conditioning, respectively); VLT (velocity related to lactate threshold) was used as a guide to determine the training intensity, 70–80% VLT; fs, fecal sample; mb, muscle biomarkers (plasma lactate and pH, CK and AST). Fatigue in the IETs was identified by the fillies’ inability to follow treadmill speed.

**Fig 2:**
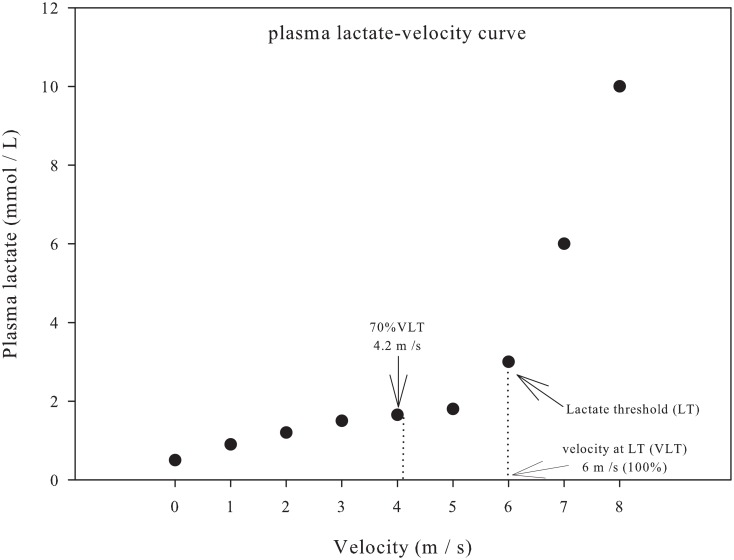
Typical relationship between plasma lactate concentration and velocity (lactate-velocity curve) in incremental exercise test (IET) involving a series of stages. Note an abrupt and exponential increase of plasma lactate concentration (deflection point-lactate threshold (LT)). The velocity at lactate threshold (VLT) has been used to elaborate the physical conditioning protocol of fillies.

An overview of the study design is shown in Fig 1. A first incremental exercise testing (IET-1) on the treadmill was conducted after an adaptation period to establish the LT from the plasma lactate concentration for each filly. The LT is determined at the point where the linear increase of the plasma lactate-velocity curve (Fig 2) shows an abrupt and exponential increase of plasma lactate concentration (deflection point). The deflection point represents the start of an imbalance between lactate production and removal/metabolism and was used as a guide to determine the related velocity (VLT) on the treadmill during the conditioning period. Thus, the initial running intensity (VLT) was set corresponding to 70% VLT. The aerobic conditioning period lasted 42 days. After this period, the fillies underwent another incremental exercise test (IET-2).

The conditioning protocol design consisted of using the treadmill and automatic walker alternately six days per week and rest on Mondays. The treadmill exercise consisted of a 5-minute warm-up walk (1.7 m s^-1^) without a slope, followed by 30 minutes of exercise with a 3% slope and speed at 70% VLT during the first two weeks and 75% VLT during the third and fourth weeks. The recovery period consisted of a 5-min walk (1.7 m s^-1^) without a slope. In the fifth and sixth weeks, the protocol consisted of a 5-minute warm-up walk (1.7 m s^-1^) with no inclination, followed by an interval workout with a 3% slope for 20 minutes, which was broken down into 4 minutes at 70% VLT, 4 minutes at 75% VLT, 4 minutes at 80% VLT, 4 minutes at 75% VLT, and 4 minutes at 70% VLT. The recovery consisted of a 5-min walk (1.7 m s^-1^) without inclination. The horses trained on the automatic walker and treadmill on alternate days; exercise on the walker consisted of a 60-min walk at 2 m s^-1^ while rotation was reversed every 10 minutes [18].

### Experimental groups and fecal collection

At the end of the adaptation period, three fillies were randomly distributed into a control group that received the same feeding regimen but did not exercise.

The remaining nine fillies were distributed according to the LT velocities that were obtained individually in the first incremental exercise test (IET-1) to balance the groups in terms of their conditioning degree. To distribute the experimental groups (exercise, L-carnitine, and chromium), a preliminary draw based on individual VLT was employed, with fillies classified into low (4 fillies between 4.23 and 4.83 m s^-1^), moderate (2 fillies between 5.00 and 5.83 m s^-1^), and high (6 fillies between 6.67 and 7.00 m s^-1^) speed. Subsequently, the fillies were randomly distributed in the experimental groups.

In total, the present study had four experimental groups: control (C, without exercise or supplementation, n = 3), exercise (E, exercise without supplementation, n = 3), L-carnitine (Car, exercise and supplementation with L-carnitine, n = 3) and chromium (Chr, exercise and supplementation with chromium, n = 3).

Fecal samples were collected immediately before IET-1 (C1-0, E1-0, Car1-0 and Chr1-0) and 48 h after IET-1 (C1-48, E1-48, Car1-48, and Chr1-48) and immediately before IET-2 (C2-0, E2-0, Car2-0 and Chr2-0) and 48 h after IET-2 (C2-48, E2-48, Car2-48, and Chr2-48).

In order to investigate the impact of acute exercise and aerobic conditioning on fecal microbiota, all fillies that underwent at the exercise were analyzed as a separate treatment (total exercise group). Thus, ET groups were formed, comprising all fillies that underwent acute exercise and aerobic conditioning (n = 9): ET1-0, ET1-48, ET2-0, and ET2-48.

Finally, to evaluate the effects of supplementation and aerobic conditioning, the samples taken at the beginning of the conditioning period (C0, E0, Car0 and Chr0) were compared with samples taken after the conditioning period (C42, E42, Car42, and Chr42).

### Fecal sampling and pH measurement

Fecal samples were collected from all twelve fillies at the same time of day directly from the rectum, via rectal palpation, by the same researcher (J.R.G. Carvalho), immediately before and 48 h after the IETs. Fecal pH was determined immediately using a calibrated industrial pH meter (Digimed—DM 22, São Paulo, Brazil) in 30 g of feces diluted in 30 mL of deionized water. Aliquots of approximately 10 g were stored in a freezer at -80°C until DNA extraction was performed.

### Blood sampling and determination of plasma and serum variables of muscle activity and body weight

Blood samples were obtained via catheter before the IET, and after reaching fatigue, and transferred to collection tubes containing sodium fluoride to determine lactate concentrations by electro-enzymatically method (YSI 2300 analyzer, YSI Inc., Yellow Springs, Ohio, USA). Venous pH was determined using a portable chemistry analyzer (i-STAT cartridges EC8 +; Heska Corporation, Fort Collins, Colorado). Blood aliquots were collected immediately before and 6 h, 12 h, and 24 h after the IETs in 10-mL tubes both with and without anticoagulant and were used to evaluate CK and AST enzyme activities by spectrophotometry (Quick Lab Chemistry Analyzer, Hameln, Germany).

### DNA extraction and sequencing

DNA was extracted from fecal samples using the FastDNA SPIN kit for soil (Mpbio^®^). DNA was quantified and qualified by spectrophotometry (NanoDrop^®^, Thermo Fisher Scientific).

The V4 region of the 16S rRNA gene was amplified in a reaction containing the following: 25 μL of Kapa 2G Fast Hot Start Ready Mix 2X (Kapa Biosystems, USA), 1.3 μL of MgCl_2_ (50 mM) (Invitrogen, USA), 1.0 μL of BSA (2 mg/mL) (Bio-Rad), 16.7 μL of nuclease free water, 2 μL of DNA and 2 μL of forward (S-D-Bact-00564-a-S-15 5′-AYTGGGYDTAAAGNG-3′) and reverse (S-D-Bact-0785-b-A-18 5′-TACNVGGGTATCTAATCC-3′) primers (10 pMol/μL) as described by Klindworth et al. (2013) [20]. The oligonucleotides used in the PCR were designed such that they contained Illumina adapters and indexes, according to the manufacturer’s recommendations. PCR was carried under the following conditions: 3 min at 94°C for initial denaturation, followed by 25 cycles of 45 s at 94°C for denaturation, 1 min at 50°C for annealing and 90 s at 72°C for extension, and finally 10 min at 72°C for a final ribbon extension before holding at 4°C. Amplicon libraries were purified with magnetic beads (Agencourt AMPure XP, Beckman Coulter Inc., Mississauga, ON) by mixing 72 μL of magnetic beads with 20 μL of the amplified libraries, and, after incubation at room temperature for 5 min, samples were washed with 80% ethanol twice and eluted in 40 μL of nuclease-free water. The purified samples were quantified in a spectrophotometer (NanoDrop, Thermo Fisher Scientific) and qualified on a 1% agarose gel. After this, the samples were diluted to 5 ng/μL. Sequencing was performed at the University of Guelph’s Advanced Analysis Centre, using an Illumina MiSeq platform (Illumina RTA v1.17.28; MCS v2.2) with the 2 x 250 V2 reagents. These data are available in the NCBI Sequence Read Archive under accession number PRJNA308944.

### Bioinformatic and statistical analyses

Bioinformatic analysis was performed using Mothur software (version 1.34.4) [21]. Good-quality sequences were classified as "operational taxonomic units" (OTUs), grouped at 97% similarity and classified based on the Ribosomal Database Classifier (RDP, March 2012) [22]. Chimera detection was performed using Uchime [23].

Sub-sampling was performed based for the sample with the minimum number of reads to reduce the errors in non-uniform sequences. To ensure sub-sampling representation, Good's coverage and rarefaction curves that represented the number of OTUs by the number of reads for each sample were evaluated. The diversity of each sample was estimated by calculating the inverse of Simpson’s index, and richness was calculated using Catchall software [24]. Bar graphs representing the relative abundance of the main phyla and genera present in each group at the different time points were generated, and the Steel-Dwass test was used to compare relative abundances between groups, controlling for multiple comparisons error. The ANOVA test was used to compare the results from Catchall and the inverse of Simpson’s index calculated for each group (p < 0.05 significance level).

The similarity between the bacterial populations in each sample was compared using a distance matrix in PHYLIP format to calculate the Yue & Clayton coefficient, which takes into account the bacterial richness and evenness (community structure), as well as the Classic Jaccard coefficient, which takes into account only bacterial richness (membership). The calculations were visually represented by dendrograms that were built using Figtree software (version 1.4.0, http://tree.bio.ed.ac.uk/). Principal coordinate analysis (PCoA) was also performed to compare sample similarities in 3 dimensions using the JMP software (SAS Institute Inc.). The similarity between community structure and membership found in samples from each group was compared using the Parsimony test and by calculating the analysis of molecular variance (AMOVA). The parsimony method is a generic test that describes whether two or more communities have the same structure.

Significant differences over time (pre- vs. post-conditioning) of the energy intake, concentrate, body weight, and plasma and serum biomarkers (lactate, pH, AST, and CK) were assessed using ANOVA followed by the Holm-Sidak test. Student's t-test for paired samples was used to determine the impact of acute exercise (baseline IETs and fatigue). Statistical power-analysis test was performed. The Pearson correlation was used to estimate the relationship between Catchall results, the inverse of Simpson’s index, CK and AST enzymatic activities, lactate concentration, and plasma and fecal pH. All analyses were performed at the 5% significance level.

## Results

### Diet and body weight

**Fig 3:**
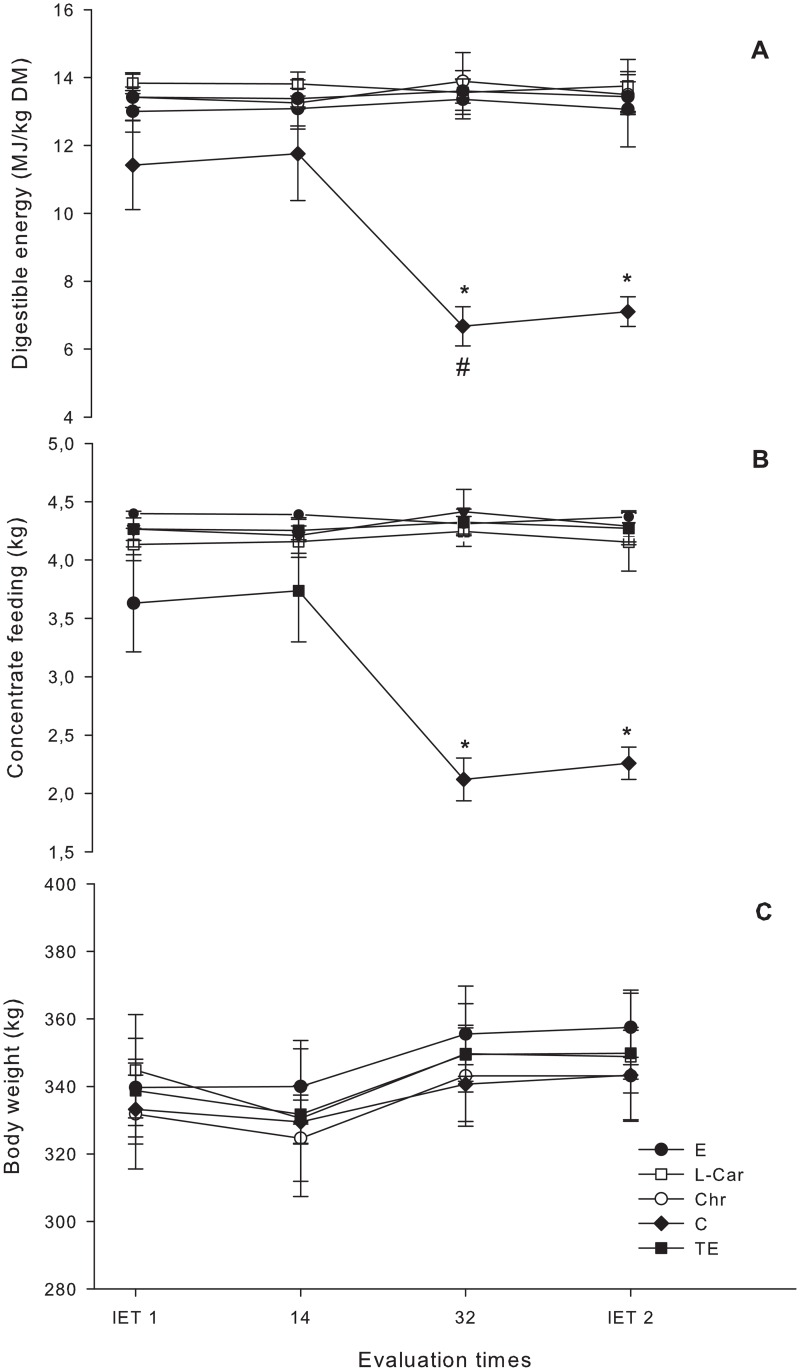
(A) Concentrate digestible energy; (B) the amount of concentrate; (C) mean body weights of fillies subjected to incremental exercise tests (IET-1 and -2) and an aerobic fitness program for 42 days. * Indicates a reduction in digestible energy and the amount of concentrate supplied to the control group compared to the other experimental groups. # indicates a reduction starting from the 32^nd^ day of conditioning with respect to the control group. The average body weight of all experimental groups remained unchanged during the trial.

Over the trial period, the control group consumed an average of 0.87 ± 0.26% of concentrate relative to body weight; in the first 14 days, the average consumption was 1.10 ± 0.01% and decreased to 0.63 ± 0.02%. The fillies undergoing the exercise program consumed 1.23 ± 0.06% body weight of concentrate. This difference was statistically significant (P = 0.020). Fig 3 and the data in S1 Table show the amounts of concentrate and digestible energy supplied and the body weights. The concentrate intake (kg) and digestible energy (Mcal) supplied to fillies in the control group started to decrease (P <0.001) in the concentrate (kg) and digestible energy (Mcal) supplied for fillies in the control group starting from the 14^th^ day after the beginning of the fitness program. Average body weight did not vary significantly (P = 0.306) in any of the experimental groups over time.

### Composition and differences in microbial communities

#### Metrics

**Fig 4:**
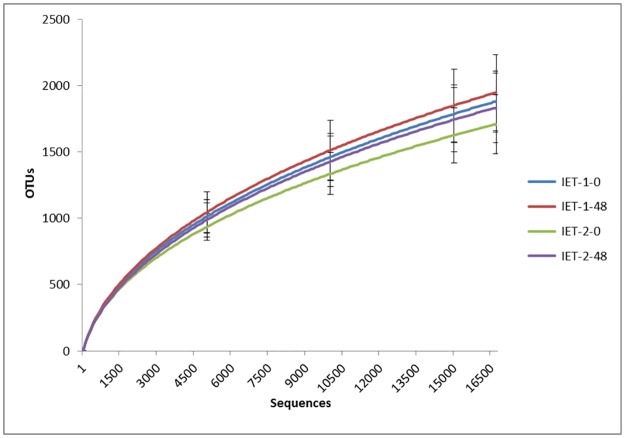
Mean values and standard deviations of rarefaction curves comparing the number of sequences and OTUs found in the DNA of fillies’ stool. Groups: IET-1-0: immediately before the first incremental test (blue); IET-1-48: 48 h after the first incremental test (red); IET-2-0: immediately before the second incremental test (green); IET-2-48: 48 h after the second incremental test (purple).

After the quality control steps and cleaning of sequences, a total of 5,224,389 sequences from 48 samples were retained (median: 98,661; SD: 38,979). A sub-sample of 16,656 sequences per sample was used to normalize the number of sequences in all samples for subsequent comparisons. The average of Good's coverage achieved after sub-sampling was 95 ± 0.9%, indicating adequate coverage. The rarefaction curves are shown in Fig 4. The median number of OTUs from the subsampled population was 1851 (min 1290 to max 2296; average 1852 ± 256).

#### Relative Abundances: effects of intense exercise and conditioning

**Fig 5:**
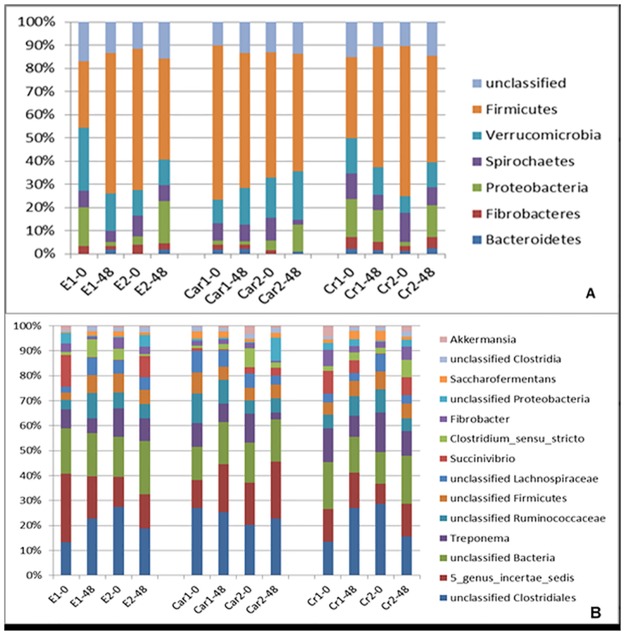
The results for the main phyla and genera found in the stool samples, with relative abundances equal to or greater than 1%. (A) Samples collected in each group: E: exercised, Car: exercised and supplemented with L-carnitine, Chr: exercised and supplemented with chromium. 1–0: Immediately before the first incremental test, 1–48: 48 h after the first incremental test, 2–0: immediately before the second incremental test, 2–48: 48 h after the second incremental test. (B) Samples collected in each group: E: exercised, Car: exercised and supplemented with L-carnitine, Chr: exercised and supplemented with chromium. 1–0: Immediately before the first incremental test, 1–48: 48 h after the first incremental test, 2–0: immediately before the second incremental test, 2–48: 48 h after the second incremental test.

Fig 5A and 5B show the phyla and genera with median relative abundances greater than 1%. The sequences obtained were classified into a total of 25 phyla, with Firmicutes (50.22%) and Verrucomicrobia (15.13%) being the most common. A total of 698 genera were assigned to 25 phyla, but only 14 had median relative abundances equal to or greater than 1%. The most abundant genus-level identifications were a Clostridiales OTU that was not classified at the genus level (17.06%) followed by “5 genus *incertae sedis*” (12.98%) from the Verrucomicrobia phylum.

#### Relative Abundances: effects of supplementation

There were no significant changes in the abundance of the main phyla and genera in response to conditioning and chromium or L-carnitine supplementation, either immediately before or 48 h after IETs (all P > 0.05). The only significant changes observed after acute exercise consisted of a decrease in the phylum Chlamydiae (P = 0.031) and in the genus *Mycobacterium* (P = 0.038) when comparing E2-0 and E2-48.

#### Population analysis–Phylotype Approach: effects of conditioning and supplementation

Table 2 shows the results from the comparison of the experimental groups that performed physical activity before and after the conditioning period and ergogenic supplementation. Membership of the control group varied significantly over time according to the results of both the parsimony and AMOVA (C0 vs. C42) tests. The exercise-only (E0 vs. E42) and exercise plus chromium supplementation (Chr0 vs. Chr42) groups underwent significant changes in their membership regardless of the statistical test applied. The AMOVA results revealed a change in the membership of the carnitine group (Car0 vs. Car42). There was no difference between chromium and carnitine supplementation compared to the exercise control group (E42 vs. Car42 and E42 vs. Chr42).

**Table 2:** Differences in the structures of the fecal microbiota communities of Mangalarga Marchador fillies that underwent a 42-days aerobic fitness program and were supplemented orally with L-carnitine and chromium as evaluated by parsimony and AMOVA analyses.

	Tests			
Comparisons	Yue & Clayton		Jaccard	
	Parsimony	AMOVA	Parsimony	AMOVA
**C0 vs. C42**	0.926	0.196	**0.031**	**0.04**
**E0 vs. E42**	0.562	0.522	**0.023**	**0.009**
**Car0 vs. Car42**	0.151	0.098	0.16	**0.047**
**Chr0 vs. Chr42**	0.29	0.147	**0.041**	**0.034**
**C42 vs. E42**	0.572	0.301	**0.014**	0.077
**C42 vs. Car42**	0.168	0.119	**0.017**	0.003
**C42 vs. Chr42**	0.765	0.097	**0.044**	**0.036**
**E42 vs. Car42**	0.572	0.86	0.938	0.771
**E42 vs. Chr42**	0.751	0.745	0.295	0.692

Control group (C0, n = 3) did not exercise; C42 = control after 42 days (did not exercise); E0 = only conditioning (before); E42 = only conditioning after 42 days; Car0 = before conditioning and L-carnitine supplementation; Car42 = conditioning and supplementation with L-carnitine after 42 days; Chr0 = before conditioning and chromium supplementation; Chr42 = conditioning and supplementation with chromium for 42 days. Effect of conditioning: E0 vs. E42; Car0 vs. Car42 and Chr0 vs. Chr42. Comparisons between control group and exercise conditioning/exercise conditioning-supplemented groups: C42 vs. E42; C42 vs. Car42 and C42 vs. Chr42. Comparisons between exercise conditioning and exercise conditioning-supplemented groups: E42 vs. Car42; E42 vs. Chr42. The numbers are P values and evaluation time with significance <0.05 are in bold.

#### Population analysis–Phylotype Approach: effects of intense exercise and conditioning

Table 3 shows the effects of IET and aerobic conditioning on the fecal microbiota communities. The results of the AMOVA test showed that acute exercise (ET1-0 vs. ET1-48 and ET2-0 vs. ET2-48) changed both, communities structure and membership. The parsimony test results also revealed significant changes in the membership comparing the beginning with the end of the trial (ET1-0 vs. ET2-48).

**Table 3:** Differences in the structures of the fecal microbiota communities of Mangalarga Marchador fillies subjected to the incremental exercise test (IET) and a 42-days aerobic fitness program as evaluated by parsimony and AMOVA analyses.

	Tests			
Comparisons	Yue & Clayton		Jaccard	
	Parsimony	AMOVA	Parsimony	AMOVA
**C1-0 vs. C1-48**	0.615	0.094	0.619	0.196
**C2-0 vs. C2-48**	0.602	0.087	0.595	0.225
**C1-48 vs. ET1-48**	1.00	**0.043**	0.229	0.05
**C2-48 vs. ET2-48**	1.00	0.238	0.199	**0.035**
**ET1-0 vs. ET1-48**	0.690	**0.027**	0.096	**0.022**
**ET2-0 vs. ET2-48**	0.219	**0.002**	**0.011**	**0.001**
**ET1-48 vs. ET2-48**	0.082	**0.003**	0.096	**0.005**
**ET1-0 vs. ET2-48**	**0.043**	0.124	**0.045**	**0.017**

C1-0 = control group before IET-1 (first incremental exercise testing) and conditioning period; C1-48 = control group 48 h after the IET-1 and before the conditioning period; C2-0 = control group before the IET-2 (second exercise testing) and after the conditioning period; C2-48 = control group 48 h after the IET-2 and after the conditioning period (C1-0, C1-48, C2-0 and C2-48, n = 3) did not exercise; ET = total exercise group: all fillies that underwent at the exercise were analyzed as a separate treatment. ET1-0 = all exercising animals before IET-1 and the conditioning period; ET1-48 = all exercising fillies 48 h after IET-1 and before the conditioning period; ET2-0 = all exercising fillies before IET-2 and after the conditioning period; ET2-48 = all exercising fillies 48 h after IET-2 and after the conditioning period (ET1-0, ET1-48, ET2-0, ET2-48, n = 9). Comparison between controls groups: C1-0 vs. C1-48; Effect of IET: C1-48 vs. ET1-48; ET1-0 vs. ET1-48 and ET2-0 vs. ET2-48. Effect of conditioning period: ET1-48 vs. ET2-48 and ET1-0 vs. ET2-48. The numbers are P values and evaluation time with significance <0.05 are in bold.

#### Dendrograms and principal coordinate analysis (PCoA)

**Fig 6:**
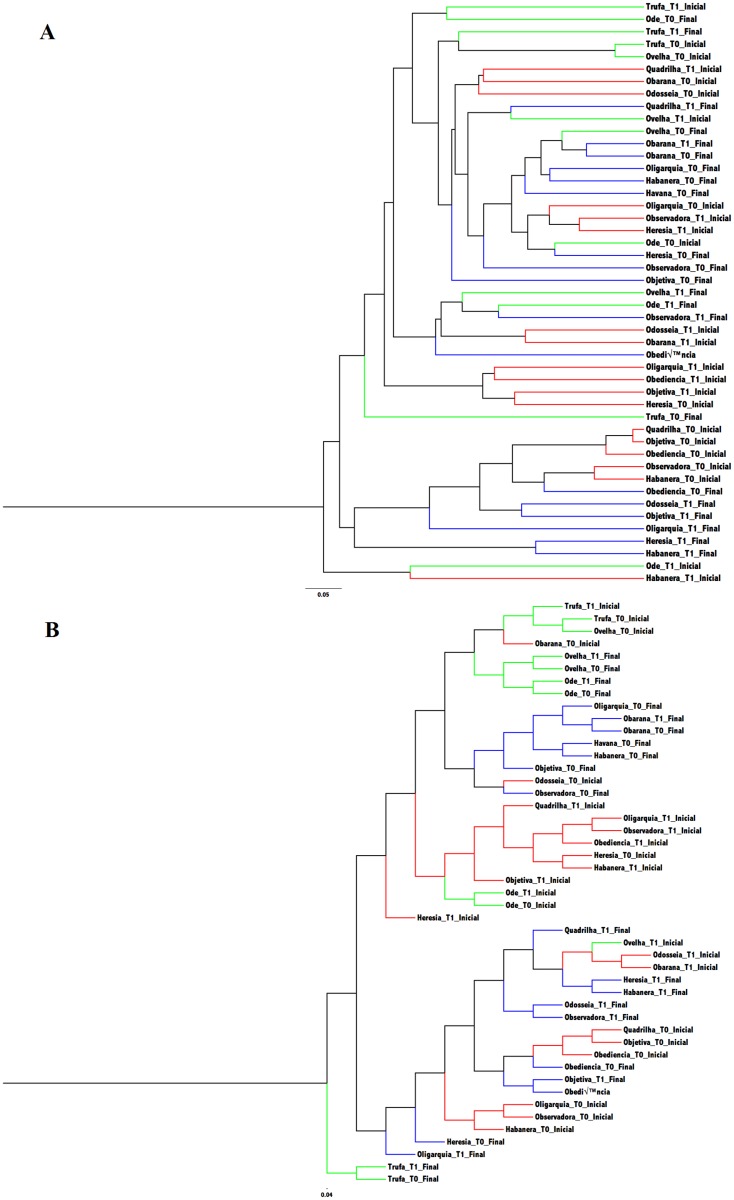
Dendrogram obtained from the analysis of the fecal microbiota of fillies subjected to the incremental exercise test on a treadmill and an aerobic fitness program. (A) Yue & Clayton analysis (red: before the conditioning period; blue: after the conditioning period, green: control). (B) Dendrogram obtained from the Jaccard index (red: before the conditioning period, blue: after the conditioning period, green: control).

**Fig 7:**
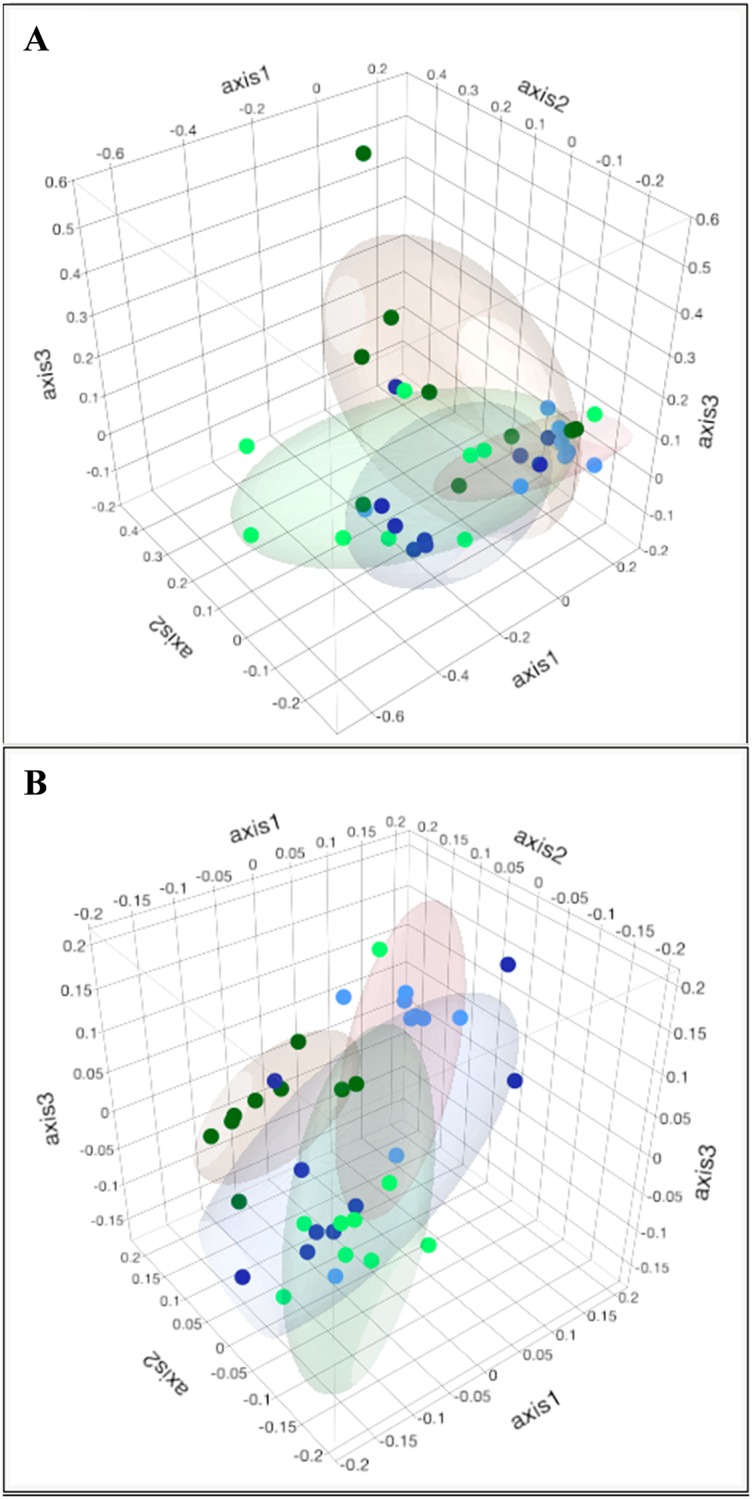
Principal component analysis (PCoA) of the fecal microbiota of fillies subjected to the incremental exercise test on a treadmill and an aerobic fitness program. (A) PCoA obtained from Yue & Clayton analysis. (B) PCoA obtained from the Jaccard index analysis. In A and B, samples are: Blue = before incremental exercise test 1 (IET-1); Light blue = 48 h after IET-1; Green = before incremental exercise test 2 (IET-2); Light green = 48 h after IET-2.

Figs 6 and 7 show dendrograms and PCoA plots, respectively, represent the similarity of microbial communities observed in each sample, demonstrating that samples collected before and after the incremental tests formed well-defined clusters in terms of membership and structure. Interestingly, in both PCoA plots, it can be observed that the samples collected at the beginning of the experiment were more similar to samples collected at the end of the conditioning period and after the last incremental test, suggesting recovery (adaptation) of the intestinal microbiota after conditioning.

No significant differences were observed in terms of the diversity (inverse Simpson's) or composition (Catchall) when comparing any of the experimental groups. The raw data values are shown in S2 Table.

### Fecal pH

The fecal pH (Table 4 and data in S3 Table) remained constant (P = 0.67) and averaged 7.69 ± 0.67 (min-max 6.41 and 8.87) in the control group (did not exercise) throughout the experimental period. There was no difference between groups E, Car and Chr. The fecal pH was significantly lower in the fillies of the ET group compared to the control group on day 21 of the trial. Immediately before IET-2, the fecal pH of the Car, Chr, E and ET and control group did not differ but decreased again 48 h after IET-2.

**Table 4:** Fecal pH of fillies subjected to incremental exercise tests (IET-1 and -2) and a 42-days aerobic fitness program associated with L-carnitine or chromium oral supplementation.

Evaluation times										
Groups	IET1-0	IET1-48	7	14	21	28	35	42	IET2-0	IET2-48
**C**	7.38±0.22	6.99±0.51	8.10±1.05	7.91±0.89	7.85±0.47	7.73±1.17	7.89±0.68	8.04±0.14	7.67±0.72	7.37±0.41
**Car**	7.62±0.45	6.88±0.34	7.16±0.53	7.18±0.72	6.58±0.04*	6.48±0.24*	6.63±0.38*	6.61±0.42*	7.38±0.17	6.80±0.44*
**Chr**	6.80±0.20	7.03±0.13	8.18±0.58	7.49±0.44	6.95±0.39	6.60±0.31	6.38±0.32*	6.68±0.43*	6.64±0.55	6.54±0.43*
**E**	6.81±0.26	7.14±0.12	7.70±0.32	7.30±0.18	7.18±0.40*	6.73±0.45*	6.88±0.34*	6.97±0.56*	7.25±0.77	6.38±0.25*
**ET**	7.08±0.49	7.01±0.22	7.68±0.61	7.32±0.45	6.90±0.38*	6.60±0.32*	6.63±0.37*	6.75±0.44*	7.09±0.59	6.57±0.38*
**F value**	2.22	0.32	1.20	0.99	5.02	3.13	6.41	5.91	1.26	3.13
**P value**	0.131	0.858	0.346	0.441	**0.008**	**0.044**	**0.003**	**0.004**	0.32	**0.004**

Control group (C, n = 3) did not exercise. L-Carnitine group (Car, n = 3). Chromium group (Chr, n = 3). Exercise group (E, n = 3): exercise without supplementation and conditioning. Exercise total group (ET, n = 9): all animals that underwent at the exercise (the incremental exercise tests and the conditioning period) were analyzed as a separate treatment. IET 1–0, IET 1–48, IET 2–0 and IET 2–48, immediately before and 48 h after the first and second IETs.

*indicates decrease in relation to the control group. Evaluation time with significance <0.05 are in bold.

### Lactate, plasma pH and muscle enzyme activities

Changes in muscle metabolism variables throughout the experimental protocol and during the two IETs are presented in Tables 5 and 6 and S4 Table. As expected, the plasma pH decreased and lactate increased significantly in all groups before and after fatigue (IET-1 and IET-2). The physical conditioning program did not affect the plasma lactate concentration at the time of fatigue (P = 0.223) for either inter-group or intra-group comparisons.

**Table 5:** Changes in the pH and plasma lactate concentration of fillies subjected to incremental exercise tests (IET-1 and IET-2) and a 42-days aerobic fitness program associated with exercise L-carnitine or chromium (Chr) oral supplementation.

Variables	Groups	Evaluation times			
		IET-1		IET-2	
		Baseline	Fatigue	Before	Fatigue
**pH**	**Car**	7.43±0.01	7.24±0.02*	7.45±0.01	7.25±0.03*
	**Chr**	7.47±0.02	7.18±0.12*	7.45±0.01	7.15±0.11*
	**E**	7.44±0.01	7.15±0.12*	7.44±0.01	7.23±0.06*
	**ET**	7.45±0.02	7.19±0.09*	7.45±0.01	7.21±0.07*
	**F value**	1.76	0.49	0.87	0.89
	**P value**	0.201	0.735	0.479	0.466
**Lactate (mmol / L)**	**Car**	0.78±0.29	20.9±1.0*	1.38±0.14	16.6±0.6*
	**Chr**	0.67±0.13	22.9±6.0*	1.42±0.03	24.6±4.8*
	**E**	0.72±0.06	22.7±4.9*	1.48±1.06	16.7±3.2*
	**ET**	0.73±0,17	22.2±4.0*	1.43±0.53	19.3±4.9*
	**F value**	0.19	0.12	0.01	2.25
	**P value**	0.890	0.940	0.998	0.127

L-Carnitine group (Car, n = 3). Chromium group (Chr, n = 3). Exercise group (E, n = 3): exercise without supplementation and conditioning. Exercise total group (ET, n = 9): all animals that underwent at the exercise (the incremental exercise tests and the conditioning period) were analyzed as a separate treatment.

*indicates increase in relation to the baseline values.

**Table 6:** Serum activities of the creatine kinase (CK) and aspartate aminotransferase (AST) enzymes of fillies subjected to incremental exercise tests (IET-1 and -2) and a 42-days aerobic fitness program associated with L-carnitine (Car) or chromium (Chr) oral supplementation.

Enzymes	Groups	Evaluation times									
		IET-1					IET-2				
		Baseline	6h	12h	24h	48h	Before	6h	12h	24h	48h
**CK (UI / L)**	**Car**	380±137	542±133	574±218	412±135	^-^	380±133	390±196	955±1108	356±158	-
	**Chr**	274±50	331±73	339±97	258±74	^-^	283±74	388±41	290±42	355±122	-
	**E**	258±50	412±97	372±85	361±101	^-^	282±37	396±122	348±13	282±56	-
	**ET**	304±96	428±129*	428±168*	344±114*	^-^	315±92	391±117	531±639	331±110	-
	**F value**	0.99	1.58	1.27	0.99		0.74	0.02	0.66	0.27	
	**P value**	0.42	0.237	0.322	0.425		0.543	1.00	0.589	0.844	
**AST (UI / L)**	**Car**	306±73	298±34	279±8	-	273±5	303±70	336±59	390±107	-	371±144
	**Chr**	269±47	264±33	289±18	-	254±12	312±57	357±71	345±63	-	301±52
	**E**	273±63	272±39	267±38	-	270±42	338±51	331±54	378±36	-	356±72
	**ET**	283±56	278±34	278±23	-	265±24	317±54	341±55	371±67	-	343±90
	**F value**	0.24	0.50	0.42		0.35	0.20	0.11	0.21		0.30
	**P value**	0.864	0.686	0.73		0.788	0.893	0.952	0.893		0.818

L-Carnitine group (Car, n = 3). Chromium group (Chr, n = 3). Exercise group (E, n = 3): exercise without supplementation and conditioning. Exercise total group (ET, n = 9): all animals that underwent at the exercise (the incremental exercise tests and the conditioning period) were analyzed as a separate treatment.

*indicates increase in relation to baseline value.

In the exercise total group (ET), the CK enzymatic activity increased (P = 0.002) by 40% 6 and 12 h after IET-1. Conversely, there was no increase (P = 0.513) in the ET group after IET-2. There was no significant difference between all experimental groups during both IET-1 and IET-2 (conditioning effect). The AST enzymatic activity remained unchanged in the experimental groups. Physical conditioning did not alter the plasma activity of either CK or AST (P = 0.292 and 0.325, respectively).

**Fig 8:**
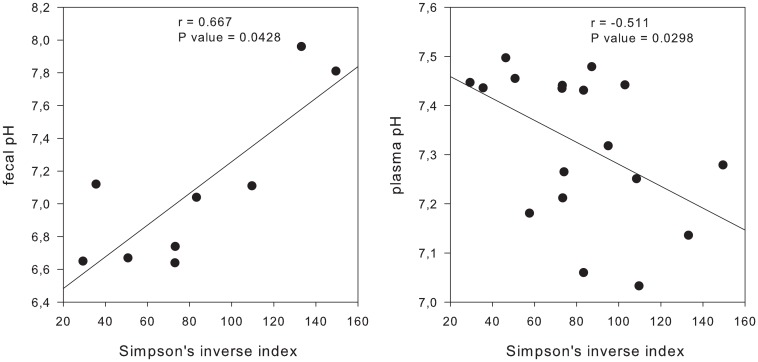
Simpson’s index and fecal pH correlation (positively) (before IET-1, without any exercise or fitness); Simpson’s index and plasma pH correlation (negatively) (immediately before and during fatigue, in the IET-1). r, correlation coefficient.

Correlations between muscle variables (plasma lactate and pH, CK and AST) and fecal pH with microbial diversity were examined by calculating Pearson correlation values. For all exercised animals, there was a positive correlation between Simpson’s index and the fecal pH before IET-1. The correlation between plasma pH and Simpson’s index (immediately before and after IET-1) was significant and negative (Fig 8).

## Discussion

Although a relationship between the intestinal microbiota and host homeostasis has become more evident, the relationship between the intestinal microbiota and physical activity is undergoing the early stages of scientific exploration. In this study, significant alterations in the microbial membership were noted in response to intense exercise, with an apparent recovery towards baseline levels as adaptation to exercise occurred. This suggests that while exercise may alter the microbiota, changes in exercise (as opposed to the amount of exercise) may be the most important factor.

Caution must be taken when interpreting the results from the comparison between groups supplemented with L-carnitine and chromium because significant differences were observed for only the Jaccard index, for the AMOVA test with the L-carnitine-supplemented group and for the AMOVA and parsimony results for chromium supplementation.

### Composition of the microbial population before and after acute exercise and aerobic conditioning

The few available studies examining the microbiota and exercise have reported conflicting results. A study in mice found that the practice of forced or voluntary exercise did not alter the two main phyla of bacteria, Firmicutes and Bacteroidetes [9]. In the same context, studies have evaluated the effects of exercise in healthy mice, and the results indicated increases in Bacteroidetes and decreases in Firmicutes [8,25]. Here, changes in these phyla were not identified, but it is important to note that the methods used in this study may favor classification of reads as Verrucomicrobia concomitantly decreasing the abundance of Bacteroidetes. Relative abundances of the main taxa reported here are in accordance with other studies that sequenced the same region (V4) of the 16S gene in fecal samples of horses [26,27]

Overall, significant differences were identified after the second incremental exercise test only in two taxa that were present at low-abundance levels (Chlamydiae and *Mycobacterium*). The phylum Chlamydie has also been reported in six healthy Irish Thoroughbred racehorses routinely undergoing active training. Each horse had been receiving their respective feed (haylage supplemented with starch concentrate) for a month [28]. Although those differences might be related to the false discovery rate, the biological meanings of these findings are not clear at this time.

The unchanged alpha diversity indices (Simpson and Catchall) associated with exercise may be due to the constant feeding regime and diet of fillies in that group. Other studies that characterized the core fecal bacterial microbiome of Irish Thoroughbred [28] or Thoroughbred racehorses receiving different feeding regimes or dietary supplementation with amylase-rich malt extract [29] found fluctuation in the alpha diversity (phylogenetic diversity, Shannon index and species evenness). In male elite professional rugby players, exercise and associated dietary extremes not only impact gut microbial diversity but also indicate that the relationship is complex [15].

It is a classical concept that beta diversity determines how phylogenetic distance is shared among samples when quantifying the number of distinct communities in a certain region [30]. In our study, the most relevant differences were observed in the PCoA plots evidenced by the formation of clusters of samples collected before and after the incremental exercise tests. Interestingly, the samples collected at the beginning of the experiment and the end of the conditioning period were similar, suggesting that the population structure of the intestinal microbiota may have adapted after the aerobic conditioning period of 42 days. The return of the population structure to pre-training levels may indicate dormancy [31] or resilience of equine intestinal microbiota-related dysbiosis [32]. This suggests that changes in exercise may exert important influences on the microbiota rather than the absolute level of exercise or specific type of exercise. From a clinical standpoint, this could be of relevance as measures to ameliorate such changes are developed or strategies are implemented to restrict other factors that might alter the microbiota (e.g., diet changes) concurrent with changes in exercise. However, it must also be considered that increasing the intensity of exercise is usually associated with increased energy intake, something that can also influence the microbiota diversity [15]. Therefore, further athletic horses studies to differentiate the effects of changes in exercise and diet are needed.

The conditioning protocol did not change the lactate concentrations of fillies. This could potentially be explained by behavioral/emotional factors presented by some fillies. For example, nervousness and difficult handling/management associated with the anticipatory effect of exercise can increase the production of catecholamines, which induce liver and muscle glycolysis/glycogenolysis and have the potential to raise lactate production [33].

### Correlation between microbial diversity and muscle variables and fecal pH

An attempt was made in the present study to correlate plasma lactate concentration, pH, and muscle enzymes activities (CK and AST) with the microbiota diversity indices with the aim of constructing a signaling network. One recent study evaluating rugby players revealed a positive correlation between certain diversity indices and plasma CK activity, suggesting that exercise can be considered an inducer of diversity in the intestine [15]. However, in the current study, CK and AST were not correlated with diversity and richness indices, and, among the studied variables, only the plasma pH, a by-product of muscular glycolysis in the first IET, was negatively correlated with Simpson’s index, suggesting that anaerobic exercise may interfere with the biodiversity in the gut. Therefore, the need to engage an anaerobic glycolytic pathway to perform exercise of a certain intensity and duration may potentially interfere with the microbiota. These interesting results contribute to our growing body of evidence on this subject, and further studies are needed to establish the causal relationships in the exercise-fatigue-change triad for the microbiota. Importantly, there was no change in intestinal transit or consistency of stool of fillies that participated in the present study.

There was a positive correlation between the microbial diversity (Simpson’s index) and fecal pH only before the first incremental exercise test. Fecal pH and its relationship with microbial populations has been studied in thoroughbred fillies during the transition from pasture to concentrate feeding. Fecal pH was relatively stable over time, but the community members of *Lactobacillus* spp. and *Streptococcus* spp., determined by culture methods, increased as the concentrate:forage (c:f) ratio also increased [2]. In contrast, fillies that exercised and were fed the same c:f ratio throughout the experimental period showed a reduction in fecal pH starting from the 14^th^ day of conditioning, but the values remained within the normal range for horses and did not suggest fecal acidosis. The minimal changes in relative abundance and beta diversity, likely induced by bacterial community changes caused by physical activity, may have contributed to this subclinical acidosis.

This work is the first to investigate a matter of fundamental importance for the equine industry. Although Mangalarga Marchador breed is used nationally and internationally (Germany, Italy, Argentina, and USA) [18] as smooth gait horses, very little is known about the fecal microbiota of this breed. Thus, this is the first study to characterize the bacterial microbiota present in the Mangalarga Marchador faeces. However, certain limitations must be noted, such as the low number of animals used in each supplemented group. Additionally, significant changes were observed in the microbiota of the control group during the experiment, despite the adjustment period. Several factors may have contributed to these changes, such as the assignment of fillies that likely had altered microbiota at the beginning of the experiment (outliers) to the control group and the dietary adjustments during the study, whose impact was more clearly observed in this group. Nevertheless, it is noteworthy that the diet provided to the exercise group was consistent over time, which allowed animals to be compared to themselves before vs. after treatment, facilitating evaluation of the unique effects of exercise on the microbiota. Finally, limitations related to next-generation sequencing, such as primers bias and the lack of resolution at lower taxonomic levels, also must be considered.

The present study was based on a small sample size of a single breed (Mangalarga Marchador). The small number of fillies reflects the difficulty of experimenting with these animals due to the high cost involved issues with handling, and, mainly, standardization of subjects of the same sex, age, and fitness condition [18]. This reality led to varied test power values for the variables assessed: 0.05 to 0.91. A limited impact of L-carnitine and chromium was noted during evaluation; however, the highly limited statistical power of these pilot data must be considered. Despite the lack of power, significant differences were observed in the Jaccard index (a measure of the microbial population in terms of the presence or absence of members) between both treatment groups and the control group. This suggests that these compounds might alter the microbiota directly or modify the response of the microbiota to exercise; however, care must be taken when interpreting these results given the lack of other demonstrable changes.

In conclusion, the findings of this study suggest that exercise and a fitness program can potentially modify the intestinal microbiota communities of fillies during the early stages of conditioning. The impact of supplementation was questionable. This study is the first to employ an experimental model of controlled intensity exercise to evaluate changes in the bacterial population of fillies, and our results suggest that the exercise-training-gut microbiota relationship in athletic horses warrants further investigation.

## Supporting Information

S1 Table Concentrate and digestible energy supplied and the body weights variables data. (XLSX)

S2 Table Inverse Simpson's and Catchall variables data. (XLSX)

S3 Table Fecal pH data. (XLSX)

S4 Table Lactate, plasma pH, and muscle enzyme data. (XLSX)

## References

[1] Costa MC, Arroyo LG, Allen-Vercoe E, Stampfli HR, Kim PT, Sturgeon A, Weese JS. Comparison of the fecal microbiota of healthy horses and horses with colitis by high throughput sequencing of the V3-V5 region of the 16S rRNA gene. PLoS One. 2012;7: e41484 10.1371/journal.pone.0041484 22859989 PMC3409227

[2] van den Berg M, Hoskin SO, Rogers CW, Grinberg A. Fecal pH and microbial populations in thoroughbred horses during transition from pasture to concentrate feeding. J Equine Vet Sci. 2013;33: 215–222.

[3] Costa MC, Stämpfli HR, Arroyo LG, Allen-Vercoe E, Gomes RG, Weese JS. Changes in the equine fecal microbiota associated with the use of systemic antimicrobial drugs. BMC Vet Res. 2015;11: 19 10.1186/s12917-015-0335-7 25644524 PMC4323147

[4] Harlow BE, Lawrence LM, Flythe MD. Diarrhea-associated pathogens, lactobacilli and cellulolytic bacteria in equine feces: Responses to antibiotic challenge. Vet Microbiol. 2013;166: 225–232. 10.1016/j.vetmic.2013.05.003 23769300

[5] Gronvold AMR, Trine ML, Sorum H, Skancke E, Yannarell AC, Mackie RI. Changes in fecal microbiota of healthy dogs administered amoxicillin. FEMS Microbiol Ecol. 2010;71: 313–326. 10.1111/j.1574-6941.2009.00808.x 20002181

[6] Weese JS, Holcombe SJ, Embertson RM, Kurtz KA, Roessner HA, Jalali M, et al Changes in the faecal microbiota of mares precede the development of post partum colic. Equine Vet J. 2014;67: 641–649. 10.1111/evj.12361 25257320

[7] Hold GL. The gut microbiota, dietary extremes and exercise. Gut. 2014; 63: 1838–1839. 10.1136/gutjnl-2014-307305 25021422

[8] Queipo-Ortuño MI, Seoane LM, Murri M, Pardo M, Gomez-Zumaquero JM, et al Gut microbiota composition in male rat models under different nutritional status and physical activity and its association with serum leptin and ghrelin levels. Plos One. 2013. 10.1371/journal.pone.0065465 PMC3665787 23724144

[9] Allen JM, Miller MEB, Pence BD, Whitlock K, Nehra V, Gaskins HR, et al Voluntary and forced exercise differentially alters the gut microbiome in C57BL/6J mice. J Appl Physiol. 2015;118: 1059–1066. 10.1152/japplphysiol.01077.2014 25678701

[10] Lambert JE, Myslicki JP, Bomhof MR, Belke DD, Shearer J, Reimer RA. Exercise training modifies gut microbiota in normal and diabetic mice. Appl Physiol Nutr Metab. 2015;40: 1–4. 10.1139/apnm-2014-0452 25962839

[11] Ferraz GC, Soares OAB, Foz NSB, Pereira MC, Queiroz-Neto A. The workload and plasma ion concentration in a training match session of high-goal (elite) polo ponies. Equine Vet J Suppl. 2010;42: 191–195. 10.1111/j.2042-3306.2010.00278.x 21059005

[12] Robergs RA, Ghiasvand F, Parker D. Reply: the wandering argument favoring a lactic acidosis. Am J Physiol Regul Integr Comp Physiol. 2006;291: R238–R239.

[13] Brancaccio P, Maffulli N, Limongelli FM. Creatine kinase monitoring in sport medicine. Br Med Bull. 2007;81: 209–230. 10.1093/bmb/ldm014 17569697

[14] O’Sullivan O, Cronin O, Clarke SF, Murphy EF, Molloy MG, Shanahan F, et al Exercise and the microbiota. Gut microbes. 2015;6: 131–136. 10.1080/19490976.2015.1011875 25800089 PMC4615660

[15] Clarke SF, Murphy EF, O'Sullivan O, Lucey AJ, Humphreys M, Hogan A, et al Exercise and associated dietary extremes impact on gut microbial diversity. Gut. 2014; 63: 1913–20. 10.1136/gutjnl-2013-306541 25021423

[16] Pagan JD, Jackson SG, Duren SE. The effect of chromium supplementation on metabolic response to exercise in thoroughbred horses. Equine Nutrition and physiology society. 1995;14: 96–101.

[17] Rivero JL, Sporleder HP, Quiroz-Rothe E, Vervuert I, Coenen M, Harmeyer J. Oral L-carnitine combined with training promotes changes in skeletal muscle. Equine Vet J. 2002;34: 269–274. 10.1111/j.2042-3306.2002.tb05431.x 12405699

[18] Fonseca MG, Rezende ASC, Jordão LR, Lage J, Almeida MLM, Andrade JM, et al Chromium or L-carnitine supplementation during an aerobic conditioning program mildly modified the energy metabolism biomarker response in Mangalarga Marchador fillies. Livest Sci. 2015;177: 165–174.

[19] Nutrient Requirements of Horses. 6 ed Washington: The National Academies Press; 2007.

[20] Klindworth A, Pruesse E, Schweer T, Peplies J, Quast C, Horn M, Glöckner FO: Evaluation of general 16S ribosomal RNA gene PCR primers for classical and next-generation sequencing-based diversity studies. Nucleic Acids Res. 2013, gks808. 10.1093/nar/gks808 PMC3592464 22933715

[21] Kozich JJ, Westcott SL, Baxter NT, Highlander SK, Schloss PD. Development of a dual-index sequencing strategy and curation pipeline for analyzing amplicon sequence data on the MiSeq Illumina sequencing platform. Appl Environ Microbiol. 2013;79: 5112–5120. 10.1128/AEM.01043-13 23793624 PMC3753973

[22] Cole JR, Wang Q, Fish JA, Chai B, McGarrell DM, Sun Y, et al Ribosomal Database Project: data and tools for high throughput rRNA analysis. Nucleic Acids Res. 2013. 10.1093/nar/gkt1244 PMC3965039 24288368

[23] Edgar RC, Haas BJ, Clemente JC, Quince C, Knight R. UCHIME improves sensitivity and speed of chimera detection. Bioinformatics. 2011;27: 2194–2200. 10.1093/bioinformatics/btr381 21700674 PMC3150044

[24] Bunge J. Estimating the number of species with CatchAll. Pac Symp Biocomput. 2011; 121–130. 21121040 10.1142/9789814335058_0014

[25] Evans CC, LePard KJ, Kwak JW, Stancukas MC, Laskowski S, Dougherty J, et al Exercise prevents weight gain and alters the gut microbiota in a mouse model of high fat diet-induced obesity. PLoS One. 2014 9(3):e92193 10.1371/journal.pone.0092193 24670791 PMC3966766

[26] Costa MC, Stämpfli HR, Allen-Vercoe E, Weese JS Development of the faecal microbiota in foals. Equine Vet J. 2015; 10.1111/evj.12532 26518456

[27] Costa MC, Silva G, Ramos RV, Staempfli HR, Arroyo LG, Kim P, Weese JS. Characterization and comparison of the bacterial microbiota in different gastrointestinal tract compartments in horses. Vet J. 2015;205: 74–80. 10.1016/j.tvjl.2015.03.018 25975855

[28] O'Donnell MM, Harris HMB, Jeffery IB, Claesson MJ, Younge B, O'Toole PW, et al The core faecal bacterial microbiome of Irish Thoroughbred racehorses. Lett Appl Microbiol. 2013;57: 492–501. 10.1111/lam.12137 23889584

[29] Proudman CJ, Hunter JO, Darby AC, Escalona EE, Batty C, Turner C. Characterisation of the faecal metabolome and microbiome of Thoroughbred racehorses. Equine Vet J. 2015;47: 580–586. 10.1111/evj.12324 25041526

[30] Jost L. Partitioning diversity into independent alpha and beta components. Ecology. 2007;88: 2427–2439. 10.1890/06-1736 18027744

[31] Lennon JT, Jones SE Microbial seed banks: the ecological and evolutionary implications of dormancy. Nature Rev Microbiol. 2011;9: 119–130. 21233850 10.1038/nrmicro2504

[32] Janabi AHD, Biddle AS, Klein D, McKeever KH. Exercise training-induced changes in the gut microbiota of Standardbred racehorses. Comp Exer Physio. 2016:12: 119–130.

[33] Ferraz GC, Teixeira-Neto AR, Mataqueiro MI, Lacerda-Neto JC, Queiroz-Neto A. Effects of intravenous administration of caffeine on physiologic variables in exercising horses. Am J Vet Res. 2008;69: 1670–1675. 10.2460/ajvr.69.12.1670 19046017

